# Efficiency and Risk Assessment of Dental Bridge Removal Tools on Implant Abutments

**DOI:** 10.3390/jfb17010033

**Published:** 2026-01-08

**Authors:** Gianmario Schierano, Domenico Baldi, Cristina Bignardi, Mara Terzini, Andrea Tancredi Lugas

**Affiliations:** 1 Department of Surgical Sciences, C.I.R. Dental School, University of Turin, Via Nizza 230, 10126 Turin, Italy; gianmario.schierano@unito.it; 2 Department of Surgical Sciences (DISC), Division of Prosthetic Dentistry, University of Genoa, Largo R. Benzi 10, 16132 Genoa, Italy; baldi.domenico@unige.it; 3 Polito^BIO^Med Lab, Politecnico di Torino, Corso Duca degli Abruzzi 24, 10129 Turin, Italy; cristina.bignardi@polito.it (C.B.); mara.terzini@polito.it (M.T.); 4 Department of Mechanical and Aerospace Engineering, Politecnico di Torino, Corso Duca degli Abruzzi 24, 10129 Turin, Italy

**Keywords:** dental implants, cement, prosthesis, fixed bridge, removal tools, efficiency

## Abstract

This study evaluated the efficiency and potential risks associated with three clinical tools for removing cement-retained implant-supported prostheses: Magnetic Mallet, sliding hammer, and Coronaflex. The tests consisted of: cementation of three-unit bridge models onto titanium abutments with different geometries using Zinc Oxide non-eugenol or Zinc Phosphate cement. Seven different geometries of three-unit bridges were tested; therefore, a total of 7 bridges × 2 luting agents × 3 tools were combined in a full factorial analysis. Five test replicates were performed for each combination, resulting in a total of 5 × 7 × 2 × 3 = 210 retrieval tests. The 70 tests regarding the Coronaflex were taken from a previously conducted experiment on the topic, using the same dental bridge models and the same experimental conditions. Efficiency was assessed by the percentage of successful removals and the maximum force recorded with a piezoelectric load cell. For temporary cementations, the sliding hammer achieved the highest retrieval rate, while the Magnetic Mallet demonstrated comparable efficiency with lower forces. Coronaflex showed lower success rates and higher forces than Magnetic Mallet. For permanent cementations, most bridges were not removable, and attempts with the sliding hammer occasionally resulted in abutment screw damage. Within the limitations of this study, the Magnetic Mallet appears to be an effective option for removing bridges cemented with temporary cement, potentially in combination with a sliding hammer for highly retentive geometries. Zinc phosphate cement should be avoided when retrievability is desired, except for abutments with very low retention capability.

## 1. Introduction

Implant-supported prosthetic rehabilitation has become a widely accepted and effective treatment modality for the replacement of missing teeth, supported by decades of clinical research, technological advances, and broad adoption in daily practice [1,2,3,4]. Increased dissemination of evidence through scientific publications and courses, coupled with improvements in implant design and instrumentation, has further expanded access to implant therapy for a wider range of clinicians and patients [5].

Concomitant with this growth, peri-implant diseases have emerged as a frequent clinical challenge. Peri-implant conditions affect a substantial proportion of treated patients, reflecting the multifactorial nature of disease onset and progression. While bacterial biofilm remains the principal etiologic factor, patient-related variables, implant and abutment design, surgical and prosthetic procedures, and the choice between screw-retained versus cement-retained restorations all contribute to risk profiles [6,7]. With specific regard to retention type, some authors have reported an association between excess residual cement and peri-implant disease in cement-retained restorations [8]; however, a recent systematic review and meta-analysis found no significant differences in peri-implantitis prevalence or bone loss when comparing screw-retained and cement-retained prostheses [9]. Thus, both retention strategies remain in use, and retrievability of cement-retained restorations continues to be clinically relevant, as it might be necessary following clinical complications, such as: the abutment screw loosening or failure, implant infection, or the substitution of the prosthesis [10,11,12,13,14]. Several factors influence the stability of the prostheses and the possibility to perform an intact removal in case of need. The cemented prostheses are influenced by the luting agent [15,16,17,18,19]; it should be strong enough to ensure the crown stability, and, simultaneously, fragile enough to consent adequate retrievability. Furthermore, the retrievability of the prostheses is strongly influenced by the geometry of the abutments, in terms of height and taper angle [15,16,17,18,19,20,21,22]. Reports in the literature highlight a stronger retention for higher abutments compared to shorter ones, and a loss of retentiveness with the increase of the abutment taper angle [23,24,25,26,27]. Although the majority of literature on the topic relies on uniaxial tensile tests, the relation between abutment geometries and retrievability was confirmed also when the disassembly is performed with impulsive forces [24,28].

When removal of cement-retained implant-supported crowns or bridges is indicated, several instrument classes are available: manual devices (e.g., sliding hammers), pneumatic/mechanical percussive systems driven by compressed air (e.g., Coronaflex), and devices generating magneto-dynamic impulses (e.g., Magnetic Mallet). Despite their widespread clinical use, limited information is available in the literature about quantitative data on impulse forces, operator variability, and the comparative performance of different instruments. Studies focus on evaluating the removability in relation to the abutment height, the taper angle of its axial walls, surface roughness, the material used for the realization of the abutment itself, and the type and thickness of the luting agent film (glass ionomer, zinc phosphate, zinc oxide, polycarboxylate, composite resin cements). Experimental data on removability are primarily obtained through uniaxial tensile tests conducted in vitro exploiting universal testing machines, without the use of clinical devices [29,30,31,32,33,34]. Few studies utilize in their experimental tests a device used in clinical procedures (Coronaflex), comparing the data with those obtained from universal testing machines, without conducting comparisons between multiple clinical devices [35,36,37]. Identifying the optimal tool for the removal of fixed prosthesis cemented on implant prosthetic abutments is essential to minimize the risks of damaging the implant prosthetic components and maximize the patients’ comfort during the procedure [38,39]. Therefore, the aim of this study was to compare three clinical retrieval tools—sliding hammer, Coronaflex, and Magnetic Mallet—under standardized conditions. The primary outcome was instrument efficiency, assessed by the percentage of successful removals and the maximum force generated during disassembly. Secondary observations included potential implant damage.

Previous studies have been conducted on the topic, comparing the maximum impulsive force and the inter- and intra-operator variability of two commonly used clinical tools: the sliding hammer and the Coronaflex [26,27]. In these studies, the impulses were applied with the two clinical instruments directly on a load cell, without interposition of any dental prosthesis, in order to estimate the maximum force that the tools could exert.

In the present work, another automatic tool—Magnetic Mallet (Meta Ergonomica, Turbigo (MI), Italy)—was investigated in order to determine (1) its inter- and intra-operator variability, and (2) its retrieval efficiency compared with the sliding hammer (Model 2305-D, ASA Dental, Bozzano Massarossa (LU), Italy) and the Coronaflex (KAVO, Biberach, Germany). By comparing instrument performance with a standardized experimental framework, the study aims to provide clinically relevant insights into efficacy and safety in the removal of cement-retained implant-supported restorations.

## 2. Materials and Methods

### 2.1. Magnetic Mallet Force Repeatability

**Figure 1:**
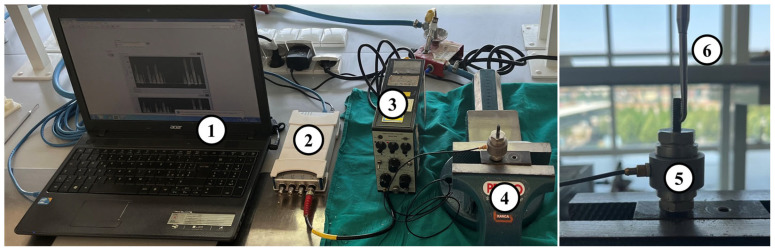
Experimental test setup: (1) laptop equipped with LabVIEW SignalExpress, (2) data acquisition system (DAQ), (3) signal amplifier, (4) bench vise, (5) piezoelectric load cell, (6) Magnetic Mallet bridge remover tip, inserted in a load-bearing screw equipped with a transverse hole.

In order to investigate the maximum forces generated by the Magnetic Mallet, the differences between the four power levels, and the intra- and inter-operator variability, of the experimental setup shown in Figure 1 was adopted. The piezoelectric load cell (type 8201, Brüel & Kjær, Nærum, Denmark, working range −20 kN–4 kN) was connected to an amplifier (type 2635, Brüel & Kjær, Nærum, Denmark) and a data acquisition board (DAQ) (NI 9234, National Instruments, Austin, TX, USA) for the signal acquisition with a sampling frequency of 51.2 kHz. The signals were recorded and monitored using a laptop equipped with the software LabVIEW SignalExpress (Version 2015, National Instruments, Austin, TX, USA). The load cell was kept still using a bench vise throughout the tests.

The impulses were directly applied to a load cell to estimate the maximum force that the tool could exert. Four operators (2 clinicians with over 20 years of experience and 2 non-clinicians) performed 50 impulses each, divided into 5 series of 10 impulses, for each of the 4 power levels of the Magnetic Mallet. Non-clinicians were included because operator variability and learnability are critical factors in assessing the instrument’s usability and efficiency. In this phase, where the primary outcome was the maximum force generated by the instrument, comparing clinicians and non-clinicians allowed evaluation of whether operator experience significantly influences performance. This approach provides insights into the instrument’s robustness and its potential for broader adoption beyond highly trained clinicians. In the following, the experienced operators will be referred to as operators A and B, and the inexperienced ones as operators C and D. A total of 800 impulses (10 impulses × 5 series × 4 operators × 4 power levels) were delivered. The series of 10 impulses was performed in a random order to avoid bias due to uncontrolled variables (e.g., operator fatigue and learning process of the inexperienced operators). All the impulses were delivered while keeping the Magnetic Mallet handle aligned with the load cell axis, in order to apply an axial force and thus be able to measure its magnitude using the single-axis load cell.

The force trend over time was recorded during each series of impulses. The peak force was computed in MATLAB 2022a (MathWorks, Inc., Natick, MA, USA) as the maximum force measured during a single impulse, and it was considered an indicator of the performance of the tool. For each group of 50 impulses performed by the same operator at the same instrument power, a coefficient of variation (i.e., the ratio between the standard deviation and the mean of the peak force) was computed and considered as an index of intra-operator variability.

### 2.2. Efficiency Comparison Between Tools

**Figure 2:**
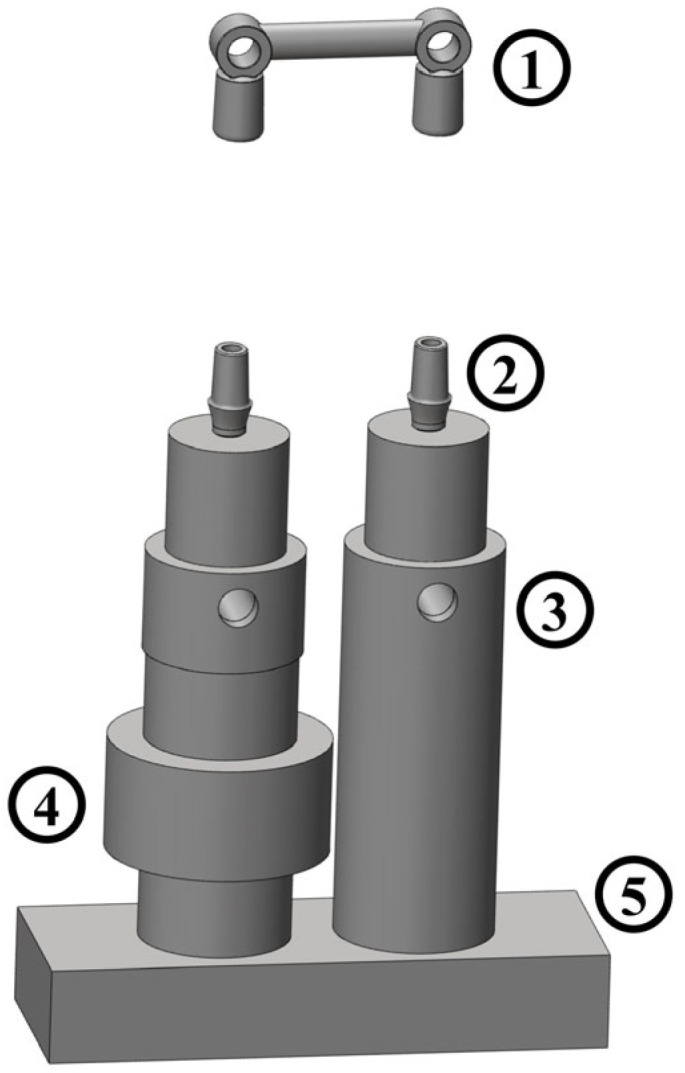
Dental bridge connection to the experimental setup: dental bridge (1); implant abutments (2); aluminum supports (3); piezoelectric load cell (4); base kept still in the bench vise (5).

Seven different dental bridge models were used in this study. Each one was composed of two copings made of noble metals (Wegold N2 alloy, Wegold Edelmetalle GmbH, Wendelstein, Germany). The 14 copings were laser-welded at the extremities of 7 noble metal alloy bars (Pagalin 2, Cendres+Métaux SA, Biel/Bienne, Switzerland), thus obtaining a biomechanical equivalent of a three-unit dental bridge. The distance between the longitudinal axes of the copings was 25 mm for every bridge dummy in order to simulate the realistic dimensions of a three-unit dental bridge. Fourteen dental implants with a diameter of 3.75 mm and a length of 13 mm (Brånemark System Mk III TiUnite RP, Nobel Biocare Italiana Srl, Milan, Italy) were inserted in as many specifically designed cylindrical aluminum supports, equipped with a threaded hole at one end to screw the dental implant and with a through hole at the other end for the connection with the experimental setup described in Figure 2.

The geometries selected for the smooth abutments and copings are reported in Table 1 in terms of height and taper angle. All abutments had an external base diameter of 5.5 mm. These configurations were chosen because they represent the most commonly used in clinical practice, based on the experience of the clinicians involved in the study. The abutments, identical to those used clinically, were manufactured using Computer Numerical Control milling from solid grade 4 titanium blocks. This process ensures high-precision machining and results in a smooth, non-porous surface. The abutments featured a height of 5–7 mm and axial wall convergence angles of 0°, 2°, or 4°, forming a truncated cone shape comparable to a natural tooth preparation.

The experimental protocol was conducted following the four steps described in [24]: (1) cementation; (2) 24 h wait to allow cement solidification; (3) prostheses retrieval; (4) abutment and coping cleaning from the residual cement. After each decementation, the luting agent was removed by copings and abutments with a dental instrument (ASA N 1700-2, Massarosa (Lu), Italy) and with isopropyl alcohol; an ultrasonic bath of 15 min was performed in a cleaning solution (Jel-Sol, Dentaltorino, Torino, Italy). Finally, the copings and abutments were washed with water, dried, and accurately checked for any luting agent residuals before the following cementation. The next cementation was performed at least 48 h after cleaning.

Two luting agents were used: a temporary zinc oxide non-eugenol cement (Temp Bond NE, Kerr Italia, Salerno, Italy) and a permanent zinc phosphate cement (Harvard Cement, Harvard Dental Company, Hoppegarten, Germany). Cementation was performed according to manufacturer instructions. Harvard Cement was mixed on a glass plate for approximately 90 s by dispensing 4–5 drops of liquid and progressively incorporating small increments of powder every 15 s, following the ratio of one dedicated scoop of powder per drop of liquid, until a workable consistency was achieved. Temp Bond NE was prepared by dispensing equal amounts of base and activator and mixing on the designated mixing pad until a homogeneous consistency was obtained. For both cements, a stainless-steel spatula was used to apply a thin layer approximately 1 mm from the margin of the coping. This technique ensured uniform distribution along the axial walls without reaching the top of the abutment, which could otherwise prevent complete seating of the restoration. Each coping was then seated on its abutment under moderate, uniform pressure for 8 min, and any excess cement was carefully removed with the appropriate spatula.

The prostheses disassembly was performed with the Magnetic Mallet and a sliding hammer. The latter is a manual tool that consists of a steel rod and a sling mass that can slide on it. The prosthesis remover tool can be screwed at each end of the rod, and the sling mass is manually moved by the operator. Thus, the applied impulsive force depends on the sliding mass speed and is strongly operator-dependent [26].

**Figure 3:**
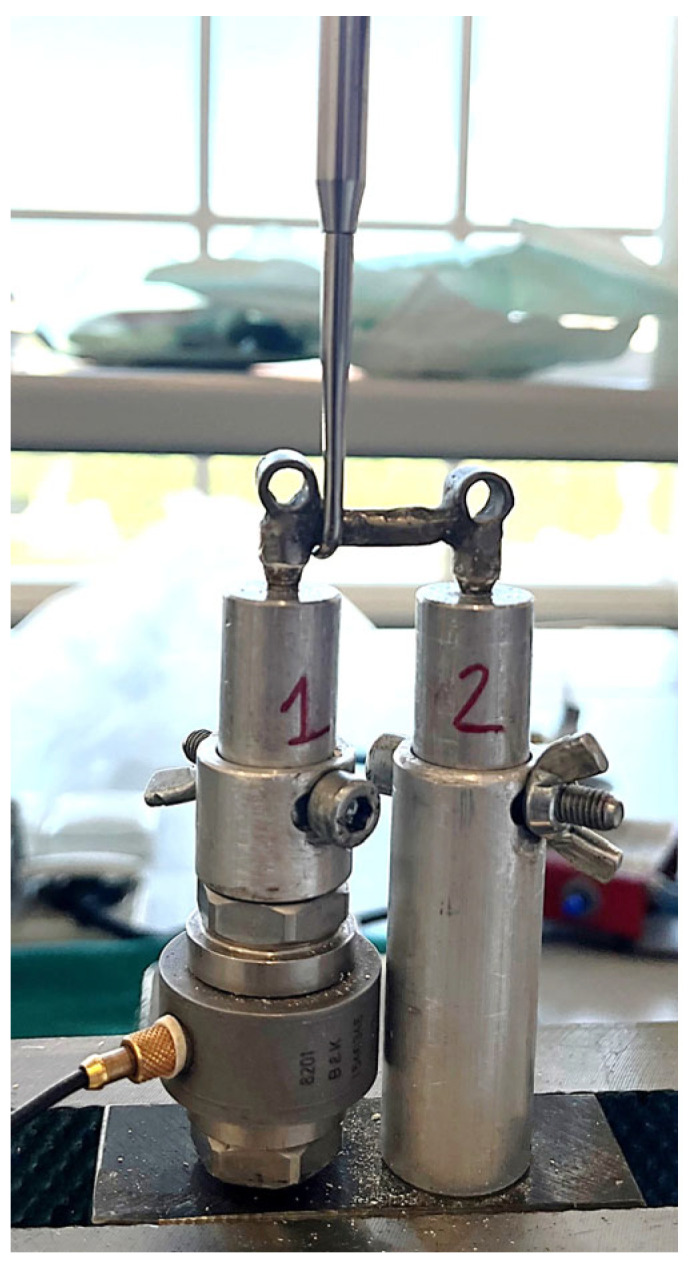
Bridge remover tip positioning during retrieval.

The same protocol was used to perform the retrievals with both instruments. The bridge remover tool was placed under the dental bridge dummy, starting from the side of the most retentive abutment, placed above the load cell (Figure 3).

The number of impulses delivered on each side of the bridge was noted down using a spreadsheet and determined as follows:

1. In case of no visible cement failure, 5 impulses were delivered on the most retentive side, followed by 5 impulses on the least retentive one, alternating the two sides until a maximum of 50 impulses.
2. In case of cement failure at any coping, the following impulses were delivered at the opposite end of the bridge until complete cement failure or until a maximum of 50 impulses were delivered in total.
3. In case of visible partial cement failure at both ends of the bridge with no complete separation between the copings and the abutments, the test continued following the protocol described in the previous two points.
4. The impulses were delivered with an interval of approximately one second between each, and a record of the location of each applied impulse was kept for all tests.

Bridges not completely removed within 50 impulses were considered non-retrievable. Given that the sliding hammer has previously been demonstrated to be a strongly operator-dependent tool [27], and that operator B was the most experienced clinician in using the Magnetic Mallet and the sliding hammer as prostheses retrieval tools, operator B was selected to perform all the bridge removals with both instruments. Five replicates were performed for each test, thus obtaining a total of 140 tests (1 operator × 2 luting agents × 2 instruments × 7 bridges × 5 replicates).

As described above, the force trend over time was recorded. The percentage of successfully removed bridges was considered as an index of the instrument performance, and the maximum force generated during the procedure was considered an indicator of the potential risks during a prosthesis removal operation. The comparison between the two devices used in this study and the Coronaflex was conducted considering the data collected in our previous study [24]. The tests in the present study were performed under identical experimental conditions and by the same operator to ensure consistency and comparability. Similarly to our previous study [24], the load cell was placed under the most retentive side of the abutment, and, therefore, only the impulses delivered in correspondence with that side were considered for the computation of the maximum peak force.

### 2.3. Statistical Analysis

To investigate the inter-operator variability, as well as the differences between the power levels of the instrument, a three-way nested analysis of variance (ANOVA) was conducted using the operator (levels: A, B, C, D), the experience (levels: experienced, not experienced), and the power (levels: 1, 2, 3, 4) as factors, and the peak force as a dependent variable. Specifically, the factor operator was nested in the factor experience. This design of the statistical analysis allows us to not take into consideration the combinations between experienced operators and the “not experienced” level of the experience factor, and vice versa, since these interactions cannot exist in the design of experiment. Considering the significant influence of the instrument’s power level and the operator, and the insignificant influence of the operator experience, it was deemed appropriate to conduct separate one-way ANOVAs for each power level, with the operator (levels: A, B, C, D) as the only factor. This choice allows for a more precise evaluation of the operator effect within each level of power, avoiding the potential for misinterpretation that could arise from pooling data across different power conditions. By analyzing each power level independently, the variability in operator performance can be more accurately assessed in the context of specific power settings, providing a clearer understanding of how the effect of the operator is influenced by the power level. A post-hoc Tukey-Kramer test was performed for the pairwise comparisons.

A Kruskal-Wallis test was conducted—factor: device; levels: Magnetic Mallet, sliding hammer, and Coronaflex—to investigate the influence of the tool used for the disassembly on the maximum forces transmitted to the load cell. Each combination of bridge and cement was investigated separately. Therefore, a total of 14 (7 bridges × 2 cements) statistical analyses were performed. Post-hoc Tuckey-Kramer tests were used for pairwise comparisons.

## 3. Results

### 3.1. Magnetic Mallet Force Repeatability

**Figure 4:**
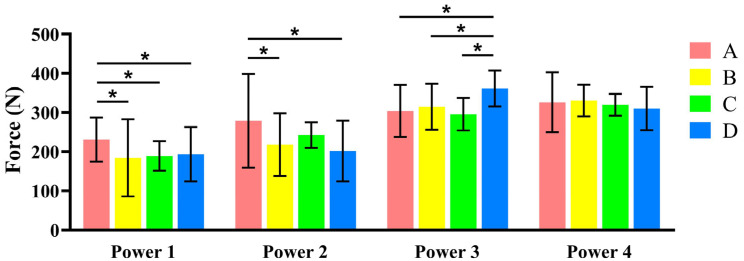
Average peak force (mean ± SD) performed by each operator (A, B, C, D) at each power level (1, 2, 3, 4) of the Magnetic Mallet. (* Significant differences *p* < 0.05). Additional data is available in the Supplementary Materials (Tables S1 and S2).

Each bar in Figure 4 shows the mean and standard deviation of the measured force computed from the 50 impulses performed by the same operator at the same power level.

Separated ANOVAs were conducted to investigate the inter-operator variability at each power of the instrument. At power 1, the forces generated by operator A were significantly higher in comparison with the ones obtained by the other three operators (*p* < 0.05). At power 2, the forces generated by operator A were significantly higher than those generated by operators B and D (*p* < 0.05) and similar to C. At power 3, all operators were similar except for D (*p* < 0.05), which generated significantly higher forces, and at power 4, there were no significant differences between the forces generated by the four operators. In all cases, the peak forces generated on the load cell were comprised in a range between 180 N and 350 N, approximately. Regarding the intra-operator variability, a coefficient of variation (CV) was computed for each 50-impulse subgroup shown in Figure 4. The CV ranged between 0.09 and 0.54. Overall, the lowest CVs were obtained at power levels 3 and 4. Power level 4 was selected because it provided the lowest intra- and inter-operator variability and generated peak forces lower than those reported for the Coronaflex in [26]. This choice ensures standardized performance while maintaining a lower force profile compared to alternative devices.

### 3.2. Efficiency Comparison Between Tools

**Figure 5:**
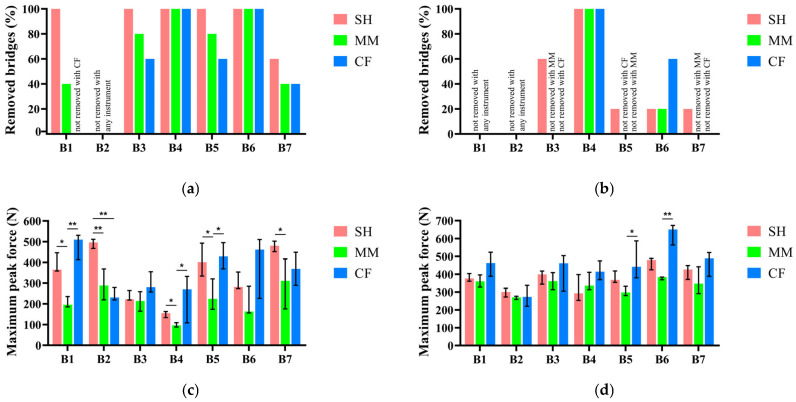
Percentage of complete bridge removals within 50 impulses. Results obtained with Temp Bond NE (**a**) and Harvard Cement (**b**). Medians and variability range (25th and 75th percentiles) of the maximum peak force measured during bridge retrieval attempts: results obtained with Temp Bond NE (**c**) and Harvard Cement (**d**). Asterisks above the bars indicate statistically significant differences (** *p* < 0.05; * *p* < 0.1). Each bar triplet represents, left to right, the data about Sliding Hammer (SH), Magnetic Mallet (MM), and Coronaflex (CF). Additional data is available in the Supplementary Materials (Tables S3–S8).

As described above, the percentage of successfully removed bridges within a certain number of impulses was considered as an efficiency index for the retrieval tools. The results of the disassembly attempts performed on bridges cemented with the temporary luting agent are reported in Figure 5a.

Each bar triplet corresponds to one of the bridges reported in Table 1.

The sliding hammer (SH) resulted as the most efficient tool according to this performance index, being able to retrieve all bridges in all tests, except for the ones with at least one abutment with 7 mm of height and 0° of taper angle, which is the most retentive among the geometries used in the study. The Magnetic Mallet (MM) achieved a similar efficiency for most bridges, with a maximum difference of 20% compared to the sliding hammer, with the exception of bridge 1, while having the same efficiency or being more efficient than the Coronaflex in all cases. Regarding the maximum force reached during the retrievals, Figure 5b shows the results obtained during the retrieval of the bridges when cemented with the temporary cement—Zinc Oxide non-eugenol—in terms of median and 25th and 75th percentiles, computed from the 5 test replicates on each bridge with each instrument. The Magnetic Mallet generated the lowest peak force with all bridges, with the only exception of bridge 2, which was the only bridge non-removable with any of the clinical instruments considered in this study. In most cases, the differences between the forces generated by Magnetic Mallet and both the sliding hammer and Coronaflex were statistically significant.

Regarding the retrieval results on the bridges cemented with the permanent luting agent (Zinc Phosphate cement), Figure 5c,d show the percentage of removed bridges and the maximum forces generated during the attempts, respectively.

Besides bridge 4, no bridge cemented with the Zinc Phosphate cement was removed in more than 60% of the cases (Figure 5c), regardless of the instrument. Moreover, the maximum force generated by the three instruments resulted in being similar for most bridges, with the only exception of bridge 5 and bridge 6, for which higher forces were registered during the removal attempts using Coronaflex (Figure 5d). Furthermore, the retention screws that connect the abutments with the implants have been damaged four times during the bridge retrieval attempts performed with the sliding hammer. These removal attempts were not considered in the force computation, and the corresponding tests are, therefore, not reported in Figure 5.

## 4. Discussion

The presented work collocates in a context of a severe lack of up-to-date evidence in the medical literature [40,41,42] and aims to make a step towards filling the knowledge gap about a crucial issue in dentistry: the retrievability of cemented dental prostheses on implants. The experimental setup provides the advantage of directly linking the bridges to be removed in a more realistic manner compared to the majority of the previous works [29,30,31,32,33,34], without the use of intermediary machines, allowing for the direct measurement of the force exerted during the removal of the bridges.

The comparison of Magnetic Mallet power settings was conducted to identify operating ranges with lower variability rather than to draw definitive conclusions about operator effects. In this dataset, intra-operator variability was lowest at power levels 3 and 4, and no inter-operator differences were detected at power level 4. The higher CVs observed at lower power settings are plausibly explained by the greater relative influence of the initial preload when positioning the removal tip on the load cell: at low power, the instrument delivers smaller impulse forces, making preload a proportionally larger contributor to the measured peak; at higher power, the impulse dominates and variability decreases. To standardize the bridge disassembly phase, power level 4 was selected, and operator B (the most experienced clinician in the team) performed all removals. This choice targeted a low intra-operator variability for both instruments and was particularly relevant because the sliding hammer is known to be operator-dependent [26,27].

The second part of the study focused on comparing the performance of the instruments in terms of retrievability, which was considered a more clinically relevant metric than the number of impulses. Although the range of luting agents used in dental practice is extensive, two representative cements were selected based on their widespread clinical use: zinc oxide as a provisional cement and zinc phosphate as a permanent cement. This choice allowed evaluation under conditions that closely replicate common clinical scenarios. Similarly, titanium abutments and platinum–palladium bridges were employed to reflect materials typically used in practice. These methodological decisions aimed to ensure that the experimental setup provided meaningful insights into the potential in vivo performance of the tested instruments.

In this study, the most common abutment geometries according to the clinicians’ expertise were included. However, in the case of particularly short abutments (shorter than 4 mm), it is necessary to create some “grooves” to increase the retention and the embrace of the prosthetic crown [43,44,45,46]. This aspect was not taken in consideration and investigated during the presented tests, which can be considered as a limit of the study.

The limit of 50 impulses was imposed in accordance with the findings in [37], in which a high discomfort was reported by patients that underwent more than 40 impulses during a prosthesis removal. Therefore, the authors of the presented study considered the application of more than 50 impulses unacceptable.

Based on the results obtained with zinc oxide non-eugenol cement, temporary cementations were generally retrieved successfully using the Magnetic Mallet, which produced the lowest forces among the three instruments tested. The sliding hammer achieved complete retrieval in all replicates, although the higher forces recorded with this manual tool may indicate an increased risk of damage to the implant–prosthetic structure. Coronaflex showed similar or lower retrieval rates compared to the Magnetic Mallet and generated higher forces, suggesting comparatively lower efficiency.

With Zinc Phosphate cement, usually adopted for permanent restorations, most bridges were never removed with Coronaflex and Magnetic Mallet and were rarely or never removed with the sliding hammer. Moreover, while retrieving the bridges cemented with Zinc Phosphate cement, during the tests performed with the sliding hammer, the retention screws of the abutments were damaged four times. Operator fatigue was not formally measured in this study and should, therefore, be considered an observational note rather than a quantified outcome. The most experienced clinician (operator B) reported greater discomfort and fatigue in the hands and arms when using the sliding hammer for the removal of bridges cemented with zinc phosphate, compared to attempts performed with automatic instruments.

This study presents limitations that should be considered when interpreting the findings. First, all removal procedures were performed by a single operator, which limits the generalizability of the results regarding operator variability and technique sensitivity. The experimental design was in vitro and did not replicate intraoral factors such as moisture, temperature, patient movement, or screw preload, which could influence instrument performance in clinical settings. Additionally, only two luting agents and seven abutment geometries were tested, whereas clinical practice involves a broader range of cements and prosthetic designs. Finally, operator fatigue was not quantitatively assessed, and its potential impact on performance remains speculative. Future research should include multi-operator trials, in vivo studies, and expanded testing across different cements and abutment configurations to validate and extend these findings.

## 5. Conclusions

This study compared different instruments for the disassembly of cement-retained implant-supported prostheses under standardized in vitro conditions. The Magnetic Mallet demonstrated high retrievability for temporary cementations while generating lower forces than the other instruments tested. For abutment geometries with greater retention (e.g., 7 mm height and 0° or 2° taper), coupling the Magnetic Mallet with a sliding hammer may be advisable. The sliding hammer achieved better retrieval results but required higher forces, which could increase the risk of damage to prosthetic components. Coronaflex showed similar or lower retrieval rates and higher forces compared to the Magnetic Mallet under the tested conditions.

Regarding the permanent cementations, the results suggest that the Zinc Phosphate cement should not be used when prostheses retrievability is desired, with the exception of abutment geometries with very low retention capability (e.g., 5 mm of height and 2 or 4 degrees of taper angle). Moreover, although able to perform a conservative disassembly of the permanently cemented prostheses in a few cases, the sliding hammer alone should not be considered an advisable tool for this task due to the risk of damaging the prostheses components.

These findings provide practical insights for clinical decision-making; however, they should be interpreted within the context of the study’s limitations: bridge removal was performed by a single operator, only two cement types and seven bridge geometries were tested, and intraoral factors such as moisture, temperature, and screw preload were not simulated. Future research—including multi-operator and in vivo studies—will be essential to confirm these observations, explore additional cement types and abutment designs, and assess operator fatigue and ergonomics.

## Figures and Tables

**Table 1:** Dental bridge geometries. The table shows the height (mm) and taper angle (°) of the two abutments of each of the seven dental bridges used in the study.

Bridge	Abutment 1	Abutment 2
B1	5 mm—0°	5 mm—0°
B2	7 mm—0°	7 mm—0°
B3	7 mm—2°	7 mm—2°
B4	5 mm—2°	5 mm—4°
B5	5 mm—0°	5 mm—4°
B6	7 mm—2°	7 mm—4°
B7	7 mm—0°	7 mm—4°

## Data Availability

The raw data supporting the conclusions of this article are publicly available at this link: https://doi.org/10.5281/zenodo.17713814 (accessed on 25 November 2025).

## Supplementary Materials

The following supporting information can be downloaded at: https://www.mdpi.com/article/10.3390/jfb17010033/s1, Tables S1–S8.
