# Supplementary material for: Efficiency and Risk Assessment of Dental Bridge Removal Tools on Implant Abutments
Source: J Funct Biomater. 2026 Jan 8;17(1):33. doi: 10.3390/jfb17010033 (PMC12843061; doi:10.3390/jfb17010033)
Supplement: Supplementary file 1 [file jfb-17-00033-s001.zip › jfb-4034513-supplementary.pdf]

Supplementary Materials:

Table S1. Average peak force (mean ± SD), measured in N, performed by each operator (A, B, C, D) at each power level (1, 2, 3, 4) of the Magnetic Mallet. Raw data from Figure 4.

	Power 1	Power 2	Power 3	Power 4
Operator A	230.84 ± 56.25	279.00 ± 119.59	304.12 ± 66.37	326.21 ± 76.44
Operator B	184.19 ± 98.72	218.24 ± 79.95	314.76 ± 58.83	330.58 ± 40.34
Operator C	189.16 ± 37.73	242.51 ± 32.67	295.73 ± 41.59	319.66 ± 27.67
Operator D	193.52 ± 69.13	202.05 ± 77.48	361.37 ± 45.81	310.33 ± 55.50

Table S2. *p*-values of the pairwise comparisons of the forces obtained at each power level of Magnetic Mallet by different operators. Raw data from Figure 4.

	Power 1	Power 2	Power 3	Power 4
A vs B	0.004	0.002	0.759	0.977
A vs C	0.014	0.126	0.866	0.927
A vs D	0.035	< 0.001	< 0.001	0.442
B vs C	0.984	0.464	0.293	0.734
B vs D	0.906	0.766	< 0.001	0.227
C vs D	0.989	0.072	< 0.001	0.817

Table S3. Number of complete bridge removals within 50 impulses, out of 5 attempts. Results obtained with Temp Bond NE. Raw data from Figure 5a.

	Sliding Hammer	Magnetic Mallet	Coronaflex
B1	5	2	0
B2	0	0	0
B3	5	4	3
B4	5	5	5
B5	5	4	3
B6	5	5	5
B7	3	2	2

Table S4. Number of complete bridge removals within 50 impulses, out of 5 attempts. Results obtained with Harvard Cement. Raw data from Figure 5b.

	Sliding Hammer	Magnetic Mallet	Coronaflex
B1	0	0	0
B2	0	0	0
B3	3	0	0
B4	5	5	5
B5	1	0	0
B6	1	1	3
B7	1	0	0

Table S5. Medians and variability range (25th and 75th percentiles) of the maximum peak force measured during bridge retrieval attempts: results obtained with Temp Bond NE. Raw data from Figure 5c.

	Sliding Hammer			Magnetic Mallet			Coronaflex		
	med	25th	75th	med	25th	75th	med	25th	75th
B1	365.0	446.6	358.9	196.2	235.7	185.8	510.2	531.0	413.8
B2	496.6	511.5	468.0	288.6	369.0	219.5	231.2	279.2	219.5
B3	222.8	264.7	218.8	214.5	259.4	164.9	280.8	355.1	257.9
B4	155.2	163.3	133.5	96.6	109.7	88.6	271.0	332.7	108.6
B5	401.7	493.3	334.3	224.6	320.6	173.8	429.5	496.0	369.4
B6	281.9	353.8	274.3	163.0	285.3	156.1	462.5	510.8	226.9
B7	480.8	502.9	452.3	311.2	416.6	175.9	369.5	449.2	289.6

Table S6. *p*-values of the pairwise comparisons of the forces obtained with each bridge by different tools: results obtained with Temp Bond NE. Raw data from Figure 5c.

	Bridge 1	Bridge 2	Bridge 3	Bridge 4	Bridge 5	Bridge 6	Bridge 7
SH vs MM	0.086	0.035	0.874	0.086	0.073	0.371	0.051
SH vs CF	0.539	0.013	0.495	0.975	0.997	0.874	0.298
MM vs CF	0.004	0.933	0.234	0.051	0.061	0.157	0.673

Table S7. Medians and variability range (25th and 75th percentiles) of the maximum peak force measured during bridge retrieval attempts: results obtained with Harvard Cement. Raw data from Figure 5d.

	Sliding Hammer			Magnetic Mallet			Coronaflex		
	med	25th	75th	med	25th	75th	med	25th	75th
B1	376.8	403.9	360.5	360.5	396.1	328.3	463.1	523.4	388.7
B2	300.5	321.7	272.6	270.7	276.7	259.6	273.6	338.1	220.0
B3	399.4	417.9	344.3	361.5	409.4	313.5	461.4	504.2	305.5
B4	293.0	398.3	253.4	336.4	410.7	313.6	414.3	475.2	369.6
B5	368.1	418.4	354.1	298.6	333.0	280.4	441.7	587.3	380.2
B6	479.8	488.9	425.0	383.3	384.2	368.7	651.2	673.3	564.4
B7	426.6	449.0	370.5	347.4	442.2	291.1	488.7	521.8	388.1

Table S8. *p*-values of the pairwise comparisons of the forces obtained with each bridge by different tools: results obtained with Harvard Cement. Raw data from Figure 5d.

	Bridge 1	Bridge 2	Bridge 3	Bridge 4	Bridge 5	Bridge 6	Bridge 7
SH vs MM	0.906	0.452	0.906	0.759	0.206	0.117	0.717
SH vs CF	0.298	0.759	0.906	0.157	0.800	0.371	0.717
MM vs CF	0.136	0.874	0.673	0.495	0.051	0.003	0.265
